# Biomedical education in the era of large language models: a paradigm shift

**DOI:** 10.1172/JCI196863

**Published:** 2025-07-15

**Authors:** Shen-Han Lee

**Affiliations:** Department of Otorhinolaryngology–Head & Neck Surgery, School of Medical Sciences, Health Campus, Universiti Sains Malaysia, and Hospital Pakar Universiti Sains Malaysia, Kubang Kerian, Kelantan, Malaysia.

The year is 2050.

The golden age of biomedical progress has ended. Science has lost its soul. Scientists do not ask “Why?” anymore. What changed?

In the early 2020s, the rise of artificial intelligence (AI) and deep learning models known as large language models (LLMs) promised to revolutionize science. Instead, the same tools created a generation of scientists who lost their ability to think critically and innovate beyond the output of their LLMs.

Jonathan, a PhD student, sits in his office staring at the computer screen. He keys in data from his latest cancer immunotherapy experiments for the LLM to analyze and watches as the model generates its conclusions. He does not question the results.

Several months later, at a conference question and answer session, a senior scientist challenges Jonathan: “Your model predicts a novel biomarker, but how do you explain its biological relevance?” Jonathan stares blankly. Flustered, he quickly types the question to the LLM on his laptop. A few elder scientists in the audience whisper to each other, “Remember when we used to think for ourselves?”

The year is 2024.

This was the year when AI was awarded two Nobel Prizes – the Physics prize for work on machine learning with artificial neural networks, the technology that powers LLMs (1); and half of the Chemistry prize, for the development of deep learning algorithms that solved a 50-year protein structure prediction challenge (2).

This was also the year when the world experienced a Cambrian explosion of LLMs. Several new models, such as GPT-4 (3), Gemini (4), Claude (5), LLaMA (6), and DeepSeek (7) were released only barely two years since ChatGPT first took the world by storm (8). Pretrained on vast amounts of text data, these deep learning models are fine-tuned for specific natural language processing tasks, such as summarizing, question answering, and classification (9).

The strength of LLMs lies in their ability to extract information across the vast scientific literature and distill it into responses that are easy to understand. Scientists can now use readily available LLMs to parse and refine their ideas, automate data analysis, summarize documents, and disseminate research findings more easily. LLMs have also been used as AI agents capable of autonomously performing tasks of a research assistant, searching the Web and documents and executing tasks related to experiments (10).

“Education is not the preparation of life; education is life itself.” This quote by 19th century educational reformer John Dewey holds true in the rapidly evolving AI landscape today. To harness the potential of LLMs, students and educators must develop the competencies and literacies to understand this technology, its limitations, and ethical concerns. For educators, LLMs can be used for curriculum development and creation of educational content, personalized learning experiences, and assessments for students (11). For students, LLMs can be used to concisely explain unfamiliar technical concepts tuned to their level of understanding, summarize the content of scientific papers, answer specific technical questions about methodology, and even provide suggestions for new research ideas based on existing literature.

By virtue of its scalability, LLMs can promote a more sustainable and inclusive biomedical workforce by breaking down barriers to education in low-resource settings. LLMs with multilingual capabilities can deliver biomedical training globally to non-native English speakers in their native languages, reducing the linguistic barrier to education (12). They can also cater to learners with disabilities through voice-to-text and text-to-speech capabilities (13).

Avoiding a dystopian AI future

Notwithstanding the immense benefits of LLMs, without a paradigm shift in biomedical education, future scientists may become overly reliant on LLMs and risk losing their ability to think critically and innovate. Therefore, the goals of post-LLM biomedical education should be to train scientists who use AI as an augmentation tool rather than a replacement for human reasoning, and cultivate thinkers rather than mere passive users of AI tools. These goals may be achieved by focusing on cross-disciplinary thinking, scientific epistemology, and the history and philosophy of science. There should also be a greater emphasis on exploring the creative process of generating ideas for scientific research to answer the question “Where do ideas come from?” (14). These skills will be crucial for a future biomedical workforce capable of merging human intelligence with AI.

Coda: the year is 2050

Biomedical science is in a golden age of creativity, innovation, and discovery. LLMs are no longer crutches for lazy thinking but tools for scientists to think deeper and work more efficiently. What changed? In the mid-2020s, biomedical education evolved to meet the needs of an AI-driven world. Education systems were redesigned to emphasize interdisciplinary knowledge and critical and creative thinking, alongside AI literacy. The use of LLMs sustainably and inclusively scaled biomedical education globally, giving birth to a new generation of biomedical scientists well equipped to thrive in an AI-powered future.

## Version 1 07/15/2025

Electronic publication
